# Oxidative stress in hypertensive children before and after 1 year of antihypertensive therapy

**DOI:** 10.1007/s00467-012-2193-x

**Published:** 2012-06-02

**Authors:** Joanna Śladowska-Kozłowska, Mieczysław Litwin, Anna Niemirska, Paweł Płudowski, Aldona Wierzbicka, Ewa Skorupa, Zbigniew T. Wawer, Roman Janas

**Affiliations:** 1 Department of Nephrology and Arterial Hypertension, The Children’s Memorial Health Institute, Warsaw, Poland; 2 Department of Research, The Children’s Memorial Health Institute, Warsaw, Poland; 3 Department of Biochemistry and Experimental Medicine, The Children’s Memorial Health Institute, Warsaw, Poland; 4 Department of Radioimmunology, The Children’s Memorial Health Institute, Warsaw, Poland

**Keywords:** Primary hypertension, Oxidative stress, Metabolic syndrome, Insulin resistance, Target organ damage, Antihypertensive therapy, Children

## Abstract

**Background:**

The relation between primary hypertension (PH), target organ damage (TOD) and oxidative stress (SOX) is not known.

**Methods:**

We assessed SOX in 86 children with PH before and after 12 months of standard non-pharmacological and pharmacological therapy based on renin-angiotensin system blockade.

**Results:**

Patients with left ventricular hypertrophy (LVH) and with carotid intima-media thickness (cIMT) >2SDS had higher thiobarbituric acid reactive substances (TBARS) concentrations in comparison to patients without LVH or with normal cIMT. Patients with metabolic syndrome (MS) had lower activity of gluthatione peroxidase, higher asymmetric dimethyloarginine (ADMA) and oxidized LDL cholesterol (oxyLDL) in comparison to patients without MS. TBARS correlated with left ventricular concentric hypertrophy, cIMT, albuminuria and SBP/24 h. ADMA and oxyLDL correlated with CRP and TG/HDL ratio. After 1 year of antihypertensive treatment blood pressure, TOD and prevalence of MS decreased. TBARS decreased and glutathione concentrations increased. The decrease of TBARS concentration correlated with the decrease of body mass index (BMI). Decrease of oxyLDL and ADMA correlated with increased insulin sensitivity, however markers of SOX did not correlate with BP decrease.

**Conclusion:**

SOX in children with PH correlates with TOD, metabolic abnormalities, changes in fat amount and improvement of insulin sensitivity, but not with BP decrease.

## Introduction

Primary hypertension (PH) is considered as a state of oxidative stress (SOX) that contributes to the development of arterio- and atherosclerosis and target organ damage (TOD) [1–4]. Abnormally elevated blood pressure causing hemodynamic insult, and typical intermediate phenotype of PH consisting of metabolic disturbances, excessive caloric intake and physical inactivity, leads to impairment of antioxidant defensive capacity [4–6]. As a consequence, reactive oxygen species cause oxidation of lipids, proteins and DNA with resultant tissue damage [7]. Furthermore, SOX is thought to be associated with adipocytokine release and renin-angiotensin-aldosterone system (RAAS) activation [8–10]. Moreover, SOX may lead to insulin resistance and is one of the elements of metabolic syndrome [11–13].

On the other hand, increased physical activity as a standard non-pharmacological intervention in hypertensive patients led to decreased reactive oxygen species generation, improved nitric oxide bioavailability and vascular function [14, 15]. Some evidence indicates that angiotensin converting enzyme inhibitors (ACEi) and angiotensin receptor blockers (ARB) may reduce oxidative stress and reactive oxygen species formation in adults with PH [16–19]. However, there are only single reports on SOX in hypertensive children [2], and there are no reports describing the relationship between SOX, TOD and antihypertensive treatment in children with PH. It is still unresolved whether increased SOX in PH is related to hemodynamic insult or to metabolic abnormalities which accompany both obesity and PH.

We hypothesized that SOX correlates with severity of PH, metabolic abnormalities and TOD in children with PH. Secondly, standard antihypertensive treatment based on non-pharmacological intervention and blockade of RAAS causes a decrease of SOX, which in turn correlates with normalization of metabolic abnormalities and regression of TOD. To test this hypothesis we assessed changes of SOX markers in relation to TOD in children with PH before and after 12 months of standard non-pharmacological and pharmacological therapy based on ACEi or ARB.

## Patients and methods

### Patients

The study adhered to the principles of the Declaration of Helsinki and was approved by the local Ethical Committee. All parents and children aged above 12 years of age provided written informed consent.

Detailed characteristics of the study group have been described previously [20, 21]. Specifically, out of 200 white, Caucasian children and adolescents admitted consecutively between 2005–2008 for investigation regarding suspected arterial hypertension, and who were not yet treated with antihypertensive drugs and in whom PH was ultimately diagnosed, 86 children aged 14.1 ± 2.4 (range: 5–17) years (20 girls and 66 boys) with untreated PH, who completed all investigative procedures, were included in the recent study. PH was diagnosed after a thorough clinical and laboratory diagnostic work-up, according to recently published recommendations [22, 23]. The exclusion criteria were: diagnosis of secondary hypertension, previous use of antihypertensive drugs, presence of any significant chronic disease (except for PH), as well as acute disease including infections in 6 weeks preceding enrollment. Patients with incomplete biochemical data were also excluded.

In all patients the diagnosis was confirmed by 24 hour ambulatory blood pressure monitoring (ABPM). ABPM was performed at the initial visit and after 12 months. Recordings lasting at least 20 hours with at least 80 % of records were considered as valid. We used the referential ABPM normative values published recently to classify patients as having normal blood pressure, prehypertension, ambulatory hypertension or severe ambulatory hypertension [24, 25]. Patients who decreased systolic blood pressure (SBP) during sleep by more than 10 % in comparison to day-time SBP values were considered as dipppers. Ambulatory hypertension was defined as mean ambulatory SBP above 95^th^ percentile and SBP load between 25 and 50 %, and severe ambulatory hypertension was defined as mean ambulatory SBP above 95^th^ percentile and SBP load above 50 %. Normotension was defined as mean ambulatory SBP below 95^th^ percentile and SBP load below 25 %. Prehypertension was defined as mean SBP below 95^th^ percentile and SBP load in the range 25 – 50 % [25].

## Methods

### Anthropometrical measurements

In all patients anthropometrical parameters, including body mass index (BMI), waist circumference (WC), waist-to-hip ratio (WHR) and waist-to-height ratio (WHTR) were measured. Obesity and being overweight were diagnosed according to International Obesity Task Force (IOTF) recommendations [26].

### Laboratory investigations

Blood samples were taken after 12 hours of fasting and were immediately sent to the laboratory. Plasma lipid peroxides were determined with the spectrofluorometric method of Yagi, and results expressed as concentration of thiobarbiturate reactive substance (TBARS). Glutathione and glutathione peroxidase activity were used as indicators of antioxidant status and were measured spectrophotometrically in erythrocytes (GSH-420(Oxis) and GPx-340(Oxis)). Concentration of oxyLDL and asymmetric dimethyloarginine (ADMA) were measured by ELISA using a commercially available kit.

Methodology of measurement of other biochemical variables, including plasma insulin, blood lipids, L-homocysteine, hsCRP and albuminuria were described previously [20, 21].

### Definition of metabolic syndrome

Because some of our patients were below 10 years of age, metabolic syndrome (MS) was diagnosed when at least 3 criteria were present (BMI ≥ 95^th^ percentile for age and gender, arterial hypertension, serum triglycerides > 110 mg/dl, fasting plasma glucose > 100 mg/dl or > 140 mg/dl at 2 h of oral glucose tolerance test and HDL cholesterol < 40 mg/dl [27]).

### Intima-media thickness measurements

Carotid and femoral superficial artery intima-media thickness (cIMT and fIMT), and wall cross-sectional area in carotid artery (WCSA) were evaluated by ultrasound according to the methodology described previously [28]. The median and standard deviation (SD) of normal values for cIMT, fIMT and WCSA were obtained from a study of 250 healthy children published elsewhere [28].

### Echocardiography (ECHO)

All ECHO examinations were performed by one examiner blinded to the severity of PH and effectiveness of treatment. ECHO measurements were performed according to American Society of Echocardiography guidelines [29]. To standardize left ventricular mass to height, left ventricular mass index (LVMi) was calculated according to the deSimone formula [30]. Left ventricular hypertrophy (LVH) was defined as an LVMi value above 95^th^ percentile for age and gender, based on the pediatric LVMi reference data of Khoury et al [31]. Relative wall thickness (RWT) was measured at end diastole as the ratio of posterior wall thickness plus interventricular septum thickness over LV internal dimension. A RWT of 0.41, which represents the 95^th^ percentile for RWT for normal children and adolescents, and the 95^th^ percentile for LVMi were used as cutoff points in evaluation of LV geometry [32].

### Antihypertensive treatment

Principles of treatment were already described previously [20, 21]. At enrollment no one patient was treated pharmacologically nor received any advice about non-pharmacological treatment. After diagnosis of PH all patients were informed about principles of non-pharmacological treatment, including lifestyle changes, increased physical activity and dietary modifications including salt avoidance, and were referred to an external consultant. All patients were encouraged to increase general physical activity up to 90 minutes daily including sport, walking, climbing stairs, etc. The character of activity (playing football, jogging, aerobic, walking, biking, etc.) was chosen by patients according to personal preferences. Dietary modifications were based on significant reduction of salt use both by patients and family members, and significant reduction of sweets.

Pharmacological treatment was immediately started in patients who had a significant TOD and/or severe ambulatory hypertension. BP was checked at the 3^rd^ and the 6^th^ month during ambulatory visits. If after 3 or 6 months office BP was still in the hypertensive range ABPM was done. In patients who still had ambulatory or severe ambulatory hypertension, pharmacotherapy was started. Finally, ABPM was performed after 12 months of treatment.

Drug therapy was based on the ACEi enalapril (0.2 – 0.3 mg/kg/day bid). Patients who had asthma or who did not tolerate ACEi were prescribed angiotensin 2 receptor type 1 blocker (ARB) losartan in a dose of 0.7 – 1 mg/kg/day in one or two daily doses.

All patients were evaluated at regular 3 months intervals during routine ambulatory, out-patient visits. Compliance to recommended lifestyle modifications was assessed by interview with patients and parents. When the antihypertensive effect was inadequate, amlodipine as the second drug was introduced and in patients treated only with lifestyle intervention, therapy with ACEi, ARB or amlodipine was started. Patients who were still hypertensive after 6 months and/or fulfilled the criteria of stage 2 hypertension based on home and casual BP measurements were prescribed metoprolol in a dose 0.5 mg/day as the third drug.

### Statistics

Because the analyzed groups included subjects of different age and gender, BMI and WC values were expressed both as absolute values and as SDS for age and gender. LVM values in g were standardized to height in meters^2.7^. The change of measured parameters was expressed as a delta value (Δ), ie the difference between measurement at 12 months and at start of treatment. Homogeneity of variance was checked with the Levene test. Continuous variables with a normal distribution were compared by the Student’s t-test for independent variables. Continuous values with non-normal distribution were compared by the Mann–Whitney U test. Comparison between groups was evaluated with an ANOVA test. Measurements taken at the start and after 12 months of treatment were compared using the Wilcoxon test. Dichotomous variables were compared using the Chi-square test, and change in prevalence during treatment was analyzed with the McNemar test. Correlation analysis was done with the Spearman test. *P* values less than 0.05 were regarded as statistically significant.

## Results

At start of treatment 67.4 % of patients were obese or overweight and their mean (±SD) BMI-SDS was +1.9 ±1.8. Metabolic syndrome was diagnosed in 13 patients (15.1 %) [20, 21] (Table 1).

**Table 1:** Descriptive demographic, clinical and laboratory data of oxidative stress and inflammatory markers at baseline and after 12 months of treatment

	Baseline	After 12 months of treatment	P
Patients with MS (%)	13 (15 %)	6 (7 %)	p = 0.07, chi^2^ = 3.27
Patients with normotension (%)	0	54 (62.8 %)	p = 0.0001, chi^2^ = 75.80
Patients with prehypertension (%)	0	10 (11.6 %)	p = 0.003, chi^2^ = 8,60
Patients with ambulatory HT (%)	50 (58.1 %)	21 (24.4 %)	p = 0.00001, chi^2^ = 27.03
Patients with severe HT (%)	36 (41.9 %)	1 (1.2 %)	p = 0.00001, chi^2^ = 33.03
Patients with LVH (%)	40 (46.5 % )	24 (27.9 % )	p = 0.003; chi^2^ =8.65
hsCRP (mg/dl)	0.86 (0.02-4.10)	0.41 (0.10-3.90)	<0.001
GSH (μmol/l)	763.4 (361.5-889.4)	778.1 (638.1-825.6)	<0.01
GPX (U/gHb)	31.6( 25.4-39.9)	31.6 (27.6-37.2)	ns
TBARS (μmol/l)	0.56 (0.14-2.73)	0.25 (0.14-1.28)	<0.001
ADMA (μmol/l)	0.54 (0.17-1.45)	0.56 (0.18-1.4)	ns
oxyLDL (mU/ml)	340.1 (104.3-1364.7)	367.5 (110.8-1409.8)	ns

Abbreviations: MS- metabolic syndrome, hsCRP – high-sensivity C-reactive protein, GSH - reduced glutathione, GPX – glutathione peroxidase activity ,TBARS – thiobarbituric acid reactive substances, ADMA – serum asymmetric dimethyloarginine, oxyLDL- oxidized low density lipoprotein cholesterol

### Relationship between SOX markers, BP, TOD and metabolic abnormalities at baseline

36 (41.9 %) patients with severe ambulatory HT had significantly lower concentrations of GSH in comparison to 50 (58.1 %) patients with ambulatory HT (748.7 (365.2-862.5) vs 773.6 (361.5 -889.4) μmol/l, *p* < 0.01). 40 patients with non-dipping status had lower values of GPX than patients with normal nocturnal fall of BP (31,2 ±1.7 vs 32.5 ±3.3 U/g Hb; *p* = 0.01).

**Fig. 1:**
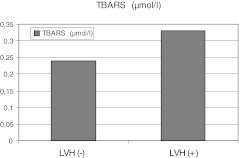
Comparison in median of thiobarbituric acid reactive substances (TBARS) concentrations between patients with- and without left ventricular hypertrophy (LVH) (p < 0.01)

40 patients with LVH had higher values of TBARS compared to 46 patients without LVH (0.33 (0.14-1.28) vs 0.24 (0.16-1.25) μmol/l, *p* = 0.02) (Figure 1). Similarly, 20 (24 %) patients with cIMT >2 SDS had greater TBARS concentration than patients showing cIMT < 2 SDS (0.36 (0.20-1.25) vs 0.26 (0.14-1.05) μmol/l, *p* < 0.01) and not significantly higher ADMA concentration (0.71 (0.25-1.37) vs 0.49 (0.17-1.45) μmol/l, *p* = 0.1) and GPX activity (32.0 (26.2-39.2) vs 31.4 (25.4-39.9) U/gHb, *p* = 0.1).

**Fig. 2:**
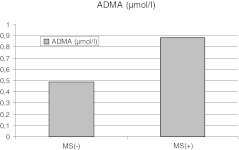
Comparison in median of asymmetric dimethyloarginine (ADMA) concentrations between patients with- and without metabolic syndrome (p < 0.01)

**Fig. 3:**
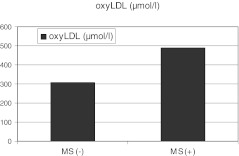
Comparison in median of oxyLDL concentrations between patients with- and without metabolic syndrome (p < 0.01)

Patients with MS had lower activity of GPX, and higher concentrations of ADMA and oxyLDL when compared to patients without MS (Table 2, Figure 2, 3).

**Table 2:** Baseline biochemical characteristics of patients with metabolic syndrome (MS[+]) and without metabolic syndrome (MS[−])

	MS[−] N = 73 (86.9 %)	MS[+] N = 13 (15.1 %)	p
HbA1C (%)	5.3 (4.2-6.8)	5.3 (4.1-6.4)	ns
TG/HDL	1.76 (0.67-4.96)	2.74 (1.65-12.9)	0.001
HOMA-IR	2.67 (0.94-9.74)	2.73 (2.04-8.77)	0.11
hsCRP (mg/dl)	0.83 (0.02-4.15)	1.2 (0.14-3.94)	ns
homocysteine(μmol/l)	9.84 ±2.52	10.7 ±1.34	ns
Uric acid (mg/dl)	5.3 ±1.2	6.8 ±1.2	<0.001
GSH (μmol/l)	761.7 (361.5-862.5)	765.1 (658.1-889.4)	ns
GPX (U/g Hb)	31.8 (25.4-39.9)	30.5 (26.2-38.7)	<0.01
TBARS (μmol/l)	0.31 (0.14-1.25)	0.26 (0.18-1.28)	ns
ADMA (μmol/l)	0.49 (0.17-1.37)	0.88 (0.21-1.45)	<0.01
oxyLDL (mU/ml)	309.0 (104.3-1255.2)	489.9 (195.7-1364.7)	0.03

Abbreviations: MS – metabolic syndrome, n – number of patients, HbA1C – glycated hemoglobin concentration, TG/HDL – triglicerydes to high density lipoprotein cholesterol ratio, HOMA-IR – homeostasis model assessment for insulin resistance, hsCRP – high-sensivity C-reactive protein, GSH - reduced glutathione, GPX – glutathione peroxidase activity ,TBARS – thiobarbituric acid reactive substances, ADMA – serum asymmetric dimethyloarginine, oxyLDL- oxidized low density lipoprotein cholesterol

TBARS concentrations correlated with LVMi, RWT, cIMT, cIMT-SDS, WCSA, urinary albumin excretion and SBP/24 h (Table 3). ADMA and oxyLDL concentrations correlated with hsCRP and TG/HDL ratio. GPX activity correlated with TG/HDL ratio and nocturnal fall of blood pressure.

**Table 3:** Correlations between parameters of oxidative stress and dependent variables at baseline (Spearman correlation analysis)

SOX parameters	Dependent variables	
TBARS	SBP/24 h	p < 0.05, r = 0.21
	LVMi	p = 0.003, r = 0.31
	RWT	p = 0.001, r = 0.35
	cIMT	p = 0.008, r = 0.29
	cIMT-SDS	p = 0.005, r = 0.30
	WCSA	p = 0.03, r = 0.26
	Urinary albumin excretion/24 h	p < 0.05, r = 0.21
	fIMT	p = 0.02, r = −0.28
	fIMT-SDS	p = 0.01 r = −0.30
GPX	nocturnal fall of SBP	P = 0.03, r = −0.235
	TG/HDL	p = 0.003, r = −0.31
	WHR	P = 0.1, r = −0.311
ADMA	WHR	P = 0.05, r = 0.24
	hsCRP	P = 0.01, r = 0.34
	GPX	p < 0.05, r = −0.24
	oxyLDL	p < 0.001, r = 0.66
	TG/HDL	p < 0.0001, r = 0.431
oxyLDL	hsCRP	P < 0.001, r = 0.662
	TG/HDL	P < 0.0001, r = 0.382
	GPX	P < 0.05, r = −0.25
	nocturnal fall of SBP	P = 0.016. r = 0.261

Abbreviations: ADMA – serum asymmetric dimethyloarginine; cIMT –carotid intima media thickness; fIMT- femoral intima media thickness; hsCRP – high-sensivity C- reactive protein; GSH - reduced glutathione; GPX – glutathione peroxidase activity; LVMi – left ventricular mass index; oxyLDL- oxidized low density lipoprotein cholesterol; RWT – relative wall thickness; SBP/24 h – mean systolic blood pressure in 24 hour-ambulatory blood pressure monitoring; TBARS – thiobarbituric acid reactive substances;TG/HDL – triglicerydes to high density lipoprotein cholesterol ratio; WCSA – wall cross sectional area; WHR – waist-hip ratio

SOX markers did not differ between overweight/obese vs normal weight patients, between patients with visceral obesity and without, and between patients with positive vs negative family history of cardiovascular disease (data not shown).

### Follow- up characteristics

**Fig. 4:**
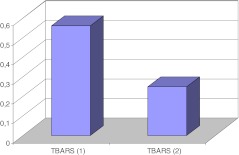
Comparison of medians of thiobarbituric acid reactive substances (TBARS) concentrations at start (TBARS1) - and after 12 months (TBARS2) of the study (p < 0.0001)

As described previously, after 1 year of antihypertensive treatment, there was a significant decrease of blood pressure, prevalence of TOD and of MS, and improvement of some metabolic abnormalities, such as a decrease of hsCRP, uric acid, total – and LDL cholesterol concentration and a decrease of visceral fat amount [20]. Compared to baseline, TBARS concentrations decreased and GSH increased (Table 1, Figure 4).

SOX markers did not correlate with blood pressure changes. 64 (73.8 %) patients who had lowered blood pressure, did not have significantly different concentrations of ADMA and oxyLDL.

Patients with regression of TOD in comparison to patients with progression/no change of TOD did not differ significantly regarding changes of SOX parameters, however patients who had decreased fIMT-SDS also had decreased oxyLDL concentrations in comparison with patients in whom fIMT-SDS increased or did not change (ΔoxyLDL: - 56.0 (−242.2-460.3) vs 82.3 (−59.1-809.5) mU/ml, *p* = 0.01). Patients in whom LVMi decreased had no significant change in oxyLDL (ΔoxyLDL: -26.5 (−626.1-810.1) vs 42.2 (−253.1-804.2) mU/ml) and ADMA (ΔADMA: -0.10 (−0.91-0.91) vs 0.18 (−0.25-0.53) μmol/l, both *p* = 0.07) compared to patients who did not have decreased LVMi.

Changes in SOX markers correlated with changes in insulin resistance and lipids. ADMA and oxyLDL concentrations decreased and GPX activity showed no significant change in patients who lowered TG/HDL ratio in comparison to patients with stabilization/increase of TG/HDL ratio (∆ ADMA: -0.16 (−0.58-0.91) vs 0.12 (−0.91-0.57) μmol/l, *p* = 0.05, ∆oxyLDL: -59.1 (−626.6-460.3) vs 100 (−295.6-810.1) mU/ml, *p* = 0.008, ∆GPX: 0.2 (−7.2-10.3) vs −0.1 (−9.1-5.3) U/gHb, *p* = 0.1). Patients in whom ADMA concentrations decreased at follow-up, had lower insulin and HbA1C concentrations, HOMA-IR, TG/HDL and LDL/HDL ratios when compared to patients with increase/stabilization of ADMA (Table 4).

**Table 4:** Comparison between patients who decreased ADMA concentrations and patients with stable/increased ADMA concentrations after 12 months of treatment

	Decrease of ADMA n = 24	Increase/stabilization of ADMA n = 20	p
∆ HOMA-IR	−0.41(−3.71-1.58)	0.31 (−2.38-1.82)	0.17
∆ HbA1C (%)	−0.1 (−1.1-4.5)	0.05 (−1.4-2)	0.03
∆TG/HDL	−0.28 ( −5.77-1.76)	0.14 (−0.90-4.41)	0.02
∆ homocysteine	−1.02 ± 3.21	1.13 ±2.29	0.02
∆ uric acid (mg/dl)	−0.38 ± 1.14	0.04 ±0.82	0.11
∆LDL/HDL	−0.61 (−1.51-1.18)	0.21 (−0.70-1.48)	0.0002
∆oxyLDL(mU/ml)	−98.95 (−626.14 – 154)	139 (−253.1-818.1)	0.0001
∆ RWT (mm)	−0.03 (−0.35-0.04)	0.007 (−0.15-0.15)	0.04
HOMA-IR at follow-up	2.12 (1.60-6.45)	3.32 (1.93-5.43)	0.008
Glu[0] (mg/dl) at follow-up	86 (71–95)	87 (75–91)	0.02
Ins[0] (mU/ml) at follow-up	10.7 ( 7,3-29)	15.7 (9–159)	0.003
LDL/HDL at follow-up	2.25 (1.50-4.11)	2.83 (1.85-3.70)	0.02
ADMA at follow-up (μmol/l)	0.505 (0.30-1.19)	0.79 (0.47 -1.37)	0.02
oxyLDL at follow-up (mU/ml)	356.12 (131–935.6)	388.7 (129.3-1409.8)	ns

Abbreviations: n - number of patients, Glu[0] – fasting plasma glucose; HOMA-IR – homeostasis model assessment for insulin resistance, HbA1C – glycated hemoglobin concentration, Ins[0] – plasma fasting insulin concentration; TG/HDL – triglicerydes to high density lipoprotein cholesterol ratio, oxyLDL- oxidized low density lipoprotein cholesterol, ADMA – serum asymmetric dimethyloarginine, RWT – relative wall thickness, glucose[0] – fasting glucose concentration, insulin [0] –fasting insulin concentration, insulin[120] – insulin concentration after 120 min. of oral glucose ingestion

The decrease of TBARS concentrations correlated with a decrease of BMI (*p* = 0.04, *r* = 0.22) but there was no statistically significant correlation with a decrease of WC (*p* = 0.1, *r* = 0.20).

The step-wise regression analysis did not reveal any significant predictors of decrease of TBARS concentrations and of increase of GSH.

## Discussion

The main finding of the study is that in newly-diagnosed hypertensive children, the severity of hypertension, TOD, metabolic abnormalities and immune activity were all associated with the magnitude of oxidative stress. The second finding is that standard antihypertensive treatment based on lifestyle modifications and/or blockade of the renin-angiotensin system caused a decrease of SOX and improved antioxidative defense. Further, the changes of SOX after 1 year of treatment correlated with regression of TOD and increased insulin sensitivity, but not directly with decrease of blood pressure.

Several reports have evaluated antioxidative defense and SOX in patients with PH and cardiovascular disease [1–6, 10, 11, 16–19]. However, in studies where classification of blood pressure status was based on clinical measurements it was found that SOX does not correlate with severity of hypertension, and even is not increased in adults with mild to moderate PH [33]. In contrast, we used ABPM to classify severity of hypertension and we found that children with severe ambulatory hypertension, ie those who are at higher risk of LVH, had significantly lower GSH concentrations and non-dippers had lower GPX activity. This may explain why, in contrast to other reports, we found significant differences regarding SOX in relation to stage of hypertension.

The corresponding finding is a strict relationship between SOX and LVH and arterial injury. Finding that SOX markers were associated with blood pressure status and TOD expressed as LVH and/or cIMT and fIMT thickening does not indicate any causation. The common pathway may be metabolic abnormalities. Baykal et al found that, despite a similar decrease of blood pressure, only ACEi and ARBs caused a significant decrease of SOX accompanied by decrease of total cholesterol, triglycerides and LDL-cholesterol concentrations [19]. It is in line with the finding that normalization of metabolic abnormalities and immune activity was the main determinant of regression of TOD in hypertensive children [20, 21]. We found that children with MS had lower GPX activity and higher ADMA and oxyLDL values in comparison to children without MS. The relationship between MS and SOX has already been described by Kelly et al, who found higher SOX in overweight children with MS compared to normal and overweight counterparts without metabolic abnormalities [34]. Siemianowicz et al revealed an association between ADMA and hsCRP levels and insulin resistance [35]. In our study we found a correlation between ADMA and markers of insulin resistance, and the decrease of ADMA concentrations correlated with a decrease of both TG/HDL ratio and direct markers of insulin resistance such as insulin, HbA1c and HOMA-IR. These relationships, observed also in adult patients, suggest that ADMA is the element that links insulin resistance and the arterio-/atherosclerotic process [11, 36, 37]. Increased ADMA concentrations in hypertensive children were found by Goonasekara et al [38]. ADMA is only an indirect marker of SOX, because dimethylarginine dimethylaminohydrolases metabolizing ADMA are inactivated by reactive oxygen species [39]. However, concentrations of ADMA are parallel to direct SOX markers as was also found in our study. Independent of vascular action, impaired nitric acid (NO) bioavailability can be related to a cellular defect in skeletal muscles, where NO regulates metabolic and contractile processes including among others, basal glucose transport [40].

It has been shown that there is relationship between genetic polymorphisms of NADPH oxidase and extent of atherosclerosis [41]. Decrease of GSH in rats caused significant increase of blood pressure, and increased antioxidative capacity diminished the hypertensive effect induced by GSH decrease [42]. However, there is only weak evidence that antioxidants may lower blood pressure [43]. One possible explanation is that SOX is secondary to hemodynamic and metabolic injury to the arterial wall and left ventricle. This is strengthened by strict relationships between changes of metabolic parameters and BMI with SOX markers. Although we found correlation between changes in BMI and changes in TBARS concentrations at follow-up, at baseline obese patients did not differ from normal-weight patients regarding SOX markers. This may be due to the narrow range of BMI in our patients. On the other hand, studies including subjects with wide BMI ranges from 18 to 45, have found that obese subjects had greater lipid peroxides concentrations in comparison with lean subjects [44]. Despite many correlations we did not find any predictors of SOX or predictors of change in SOX during treatment, which suggests that there are other modifying factors linking hypertension, metabolic abnormalities and TOD.

The main limitation of our study is the relatively low number of patients. Moreover, due to ethical reasons, we could not separate the effects of antihypertensive drugs and non-pharmacological treatment, because healthy nutrition and regular physical activity were recommended for all patients. The other limitation of our study is its observational design and lack of normotensive and BMI-matched control group. Therefore, although we found significantly increased oxidative stress in hypertensive children, and especially among children with LVH and arterial injury, we did not find a causative mechanism or explain the influence of obesity and hypertension, separately. On the other hand, the link between increased insulin sensitivity and SOX reduction underlines the importance of non-pharmacological treatment, first of all based on an increase of physical activity.

## Conclusions

We have shown the relation between oxidative stress and the severity of hypertension, TOD and metabolic disorders typical for PH. Decrease of SOX correlated with regression of TOD and normalizations of metabolic abnormalities and immune activity, but not with hypotensive effect of treatment. This finding underlines the importance of immuno-metabolic factors in the pathogenesis of hypertensive TOD and indicates that antihypertensive treatment should be focused both on hypotensive effects and metabolic abnormalities.

## References

[1] Watanabe T, Yasunari K, Nakamura M, Maeda K (2006). Carotid artery intima-media thickness and reactive oxygen species formation by monocytes in hypertensive patients. J Human Hypertens.

[2] Turi S, Friedman A, Bereczki C, Papp F, Kovàcs J, Karg E, Németh I (2003). Oxidative stress in juvenile essential hypertension. J Hypertens.

[3] Jarvisalo MJ, Putto-Laurila A, Jartti L, Lehtimäki T, Solakivi T, Rönnemaa T, Raitakari OT (2005). Carotid artery intima-media thickness in children with type 1 diabetes. Diabetes.

[4] Russo C, Olivieri O, Girelli D, Faccini G, Zenari ML, Lombardi S, Corrocher R (1998). Anti-oxidant status and lipid peroxidation in patients with essential hypertension. J Hypertens.

[5] Blankenberg S, Rupprecht HJ, Bickel C, Torzewski M, Hafner G, Tiret L, Smieja M, Cambien F, Meyer J, Lackner KJ, Investigators AtheroGene (2003). Glutathione peroxidase 1 activity and cardiovascular events in patients with coronary artery disease. N Eng J Med.

[6] Pedro-Botet J, Covas MI, Martin S, Rubies-Prat J (2000). Decreased endogenous antioxidant enzymatic status in essential hypertension. J Hum Hypertens.

[7] Valko M, Leibfritz D, Moncol J, Cronin MT, Mazur M, Telser J (2007). Free radicals and antioxidants in normal physiological functions and human disease. Int J Biochem Cell Biol.

[8] Evans JL, Goldfine ID, Maddux BA, Grodsky GM (2003). Are oxidative stress activated signaling pathways mediations of insulin resistance and beta cell dysfunction?. Diabetes.

[9] Sinaiko AR, Steinberger J, Moran A, Prineas RJ, Vessby B, Basu S, Tracy R, Jacobs DR (2005). Relation of body mass index and insulin resistance to cardiovascular risk factors, inflamatory factors, and oxidative stress during adolescence. Circulation.

[10] Kristal B, Shutz-Swirski R, Chezar J, Manaster J, Levy R, Shapiro G, Weissman I, Shasha SM, Sela S (1998). Participation of peripheral polymorphonuclear leukocytes in patients with essentials hypertension. Am J Hypertens.

[11] Perticone F, Sciacqua A, Maio R, Perticone M, Galiano Leone G, Bruni R, Di Cello S, Pascale A, Talarico G, Greco L, Andreozzi F, Sesti G (2010). Endothelial dysfunction, ADMA and insulin resistance in essentials hypertension. Int J Cardiol.

[12] Rudich A, Tirosh A, Potashnik R, Hemi R, Kanety H, Bashan N (1998). Prolonged oxidative stress impairs insulin-induced GLUT-4 translocation in 3 T3-L1 adipocytes. Diabetes.

[13] Park K, Gross M, Lee DH, Holvoet P, Himes JH, Shikany JM, Jacobs DR (2009). Oxidative stress and insulin resistance. The coronary artery risk development in young adults study. Diabetes.

[14] Melikoglu MA, Kaldirimci M, Katkat D, Sen I, Kaplan I, Senel K (2008). The effect of regular long term training on antioxidant enzymatic activities. J Sports Med Phys Fitness.

[15] Whyte JJ, Laughlin MH (2010). The effects of acute and chronic exercise on the vasculature. Acta Physiol.

[16] Yagi S, Morita T, Katayama S (2004). Combined treatment with an AT1 receptor blocker and angiotensin converting enzyme inhibitor has an additive effect on inhibiting neointima formation via improvement of nitric oxide production and suppression of oxidative stress. Hypertens Res.

[17] Ono H, Minatoguchi S, Watanabe K, Yamada Y, Mizukusa T, Kawasaki H, Takahashi H, Uno T, Tsukamoto T, Hiei K, Fujiwara H (2008). Candesartan decreases carotid intima-media thickness by enhancing nitric oxide and decreasing oxidative stress in patients with hypertension. Hypertens Res.

[18] Yasunari K, Maeda K, Watanabe T, Nakamura M, Yoshikawa J, Asada A (2004). Comparative effects of valsartan versus amlodipine on left ventricular mass and reactive oxygen species formation by monocytes in hypertensive patients with left ventricular hypertrophy. J Am Coll Cardiol.

[19] Baykal Y, Yilmaz MI, Celik T, Gok F, Rehber H, Akay C, Kocar IH (2003). Effects of antihypertensive agents, alpha receptor blockers, beta blockers, angiotensin-converting enzyme inhibitors, angiotensin receptor blockers and calcium channel blockers, on oxidative stress. J Hypertens.

[20] Litwin M, Niemirska A, Śladowska-Kozłowska J, Wierzbicka A, Janas R, Wawer ZT, Wiśniewski A, Feber J (2010). Regression of target organ damage in children and adolescents with primary hypertension. Pediatr Nephrol.

[21] Śladowska-Kozłowska J, Litwin M, Niemirska A, Wierzbicka A, Wawer ZT, Janas R (2011). Change in left ventricular geometry during antihypertensive treatment in children with primary hypertension. Pediatr Nephrol.

[22] National High Blood Pressure Education Program Working Group on High Blood Pressure in Children and Adolescents (2004) The Fourth Report on Diagnosis, Evaluation and Treatment of High Blood Pressure in Children and Adolescents Pediatrics 114:555–576 15286277

[23] Lurbe E, Cifkova R, Cruickshank JK, Dillon MJ, Ferreira I, Invitti C, Kuznetsova T, Laurent S, Mancia G, Morales-Olivas F, Rascher W, Redon J, Schaefer F, Seeman T, Stergiou G, Wühl E, Zanchetti A, European Society of Hypertension (2009). Management of high blood pressure in children and adolescents: recommendations of the European society of hypertension. J Hypertens.

[24] Wühl E, Witte K, Soergel M, Mehls O, Shaefer F, German Working Group on Pediatric Hypertension (2002). Distribution of 24-h ambulatory blood pressure in children: normalized reference values and role of body dimensions. J Hypertens.

[25] Urbina E, Alpert B, Flynn J, Hayman L, Harshfield GA, Jacobson M, Mahoney L, McCrindle B, Mietus-Snyder M, Steinberger J, Daniels S, American Heart Association Atherosclerosis, Hypertension, and Obesity in Youth Committee (2008). Ambulatory blood pressure monitoring in children and adolescents recomendations for standard assessment. A scientific statement from the American heart association atherosclerosis, hypertension, and obesity in youth committee of the council on cardiovascular disease in the young and the council for high blood pressure research. Hypertension.

[26] Cole TJ, Bellizzi MC, Flegal KM, Dietz WH (2000). Establishing a standard definition for child overweight and obesity worldwide: international study. BMJ.

[27] Litwin M, Śladowska J, Antoniewicz J, Niemirska A, Wierzbicka A, Daszkowska J, Wawer ZT, Janas R, Grenda R (2007). Metabolic abnormalities, insulin resistance and metabolic syndrome in children with primary hypertension. Am J Hypertens.

[28] Jourdan C, Wühl E, Litwin M, Fahr K, Trelewicz J, Jobs K, Schenk JP, Grenda R, Mehls O, Tröger J, Schaefer F (2005). Normative values of intima-media thickness and distensibility of large arteries in healthy adolescents. J Hypertens.

[29] Devereux RB, Alonso DR, Lutas EM, Gottlieb GJ, Campo E, Sachs I, Reichek N (1986). Echocardiographic assessment of left ventricular hypertrophy: comparison to necropsy findings. Am J Cardiol.

[30] de Simone G, Daniels SR, Devereux RB, Meyer RA, Roman MJ, de Divitiis O, Alderman MH (1992). Left ventricular mass and body size in normotensive children and adults: assessment of allometric relations and impact of overweight. J Am Coll Cardiol.

[31] Khoury PR, Mitsnefes M, Daniels SR, Kimball TR (2009). Age-specific reference intervals for indexed left ventricular mass in children. J Am Soc Echo.

[32] Daniels SR, Meyer RA, Liang Y, Bove KE (1998). Echocardiographically determined left ventricular mass index in normal children, adolescents and young adults. J Am Coll Cardiol.

[33] Redón J, Oliva MR, Tormos C, Giner V, Chaves J, Iraki A, Sáez GT (2003). Antioxidant activities and oxidative stress byproducts in human hypertension. Hypertension.

[34] Kelly AS, Steinberger J, Kaiser DR, Olson TP, Bank AJ, Dengel DR (2006). Oxidative stress and adverse adipokine profile characterize the metabolic syndrome in children. J Cardiometab Syndr.

[35] Siemianowicz K, Gmiński J, Francuz T, Wójcik A, Posielezna B (2006). Activity of antioxidant enzymes in children from families at high risk of premature coronary heart disease. Scand J Clin Lab Invest.

[36] Sydov K, Mondon CE, Cooke JP (2005). Insulin resistance: potential role of the endogenous nitric oxide synthase inhibitor ADMA. Vasc Med.

[37] Stühlinger M, Abbasi F, Chu JW, Lamendola C, McLaughlin TL, Cooke JP, Reaven GM, Tsao PS (2002). Relationship between insulin resistance and an endogenous nitric oxide synthase inhibitor. J Am Med Assoc.

[38] Goonasekera CD, Rees DD, Woolard P, Frend A, Shah V, Dillon MJ (1997). Nitric oxide synthase inhibitors and hypertension in children and adolescents. J Hypertens.

[39] Palm AJP, Onozato ML, Luo Z, Wilcox CS (2007). Dimethylarginine dimethylaminohydrolase (DDAH): expression, regulation, and function in the cardiovascular and renal systems. Am J Physiol Heart Circ Physiol.

[40] Kapur S, Bedard S, Marcotte B, Cote CH, Marette A (1997). Expression of nitric oxide synthase in skeletal muscle: a novel role for nitric oxide as a modulator of insulin action. Diabetes.

[41] Moreno MU, San José G, Fortuño A, Beloqui O, Díez J, Zalba G (2006). The C242T CYBA polymorphism of NADPH oxidase is associated with essential hypertension. J Hypertens.

[42] Vaziri ND, Wang XQ, Oveisi F, Rad B (2000). Induction of oxidative stress by glutathione depletion causes severe hypertension in normal rats. Hypertension.

[43] Touyz RM (2004). Reactive oxygen species, vascular oxidative stress, and redox signaling in hypertension: what is the clinical significance?. Hypertension.

[44] Brown LA, Kerr CJ, Whiting P, Finer R, McEneny J, Ashton T (2009). Oxidant stress in healthy normal-weight, overweight and obese individuals. Obesity.

